# Impact of body mass index on outcomes after lumbar spine surgery

**DOI:** 10.1038/s41598-023-35008-8

**Published:** 2023-05-15

**Authors:** Koji Nakajima, Junya Miyahara, Nozomu Ohtomo, Kosei Nagata, So Kato, Toru Doi, Yoshitaka Matsubayashi, Yuki Taniguchi, Naohiro Kawamura, Akiro Higashikawa, Yujiro Takeshita, Masayoshi Fukushima, Takashi Ono, Nobuhiro Hara, Seiichi Azuma, Hiroki Iwai, Masahito Oshina, Shurei Sugita, Shima Hirai, Kazuhiro Masuda, Sakae Tanaka, Yasushi Oshima

**Affiliations:** 1 Department of Orthopaedic Surgery, Faculty of Medicine, The University of Tokyo, 7-3-1 Hongo, Bunkyo-Ku, Tokyo, 113-8655 Japan; 2 University of Tokyo Spine Group (UTSG), Tokyo, Japan; 3 Department of Spine and Orthopedic Surgery, Japanese Red Cross Medical Center, Tokyo, Japan; 4 Department of Orthopedic Surgery, Kanto Rosai Hospital, Kanagawa, Japan; 5 Department of Orthopedic Surgery, Yokohama Rosai Hospital, Kanagawa, Japan; 6 Spine Center, Toranomon Hospital, Tokyo, Japan; 7 Department of Spinal Surgery, Japan Community Health-Care Organization Tokyo Shinjuku Medical Center, Tokyo, Japan; 8 Department of Orthopedic Surgery, Japanese Red Cross Musashino Hospital, Tokyo, Japan; 9 Department of Orthopedic Surgery, Saitama Red Cross Hospital, Saitama, Japan; 10 Department of Orthopedic Surgery, Iwai Orthopaedic Medical Hospital, Tokyo, Japan; 11 Spine Center, NTT Medical Center Tokyo, Tokyo, Japan; 12 Department of Orthopedic Surgery, Tokyo Metropolitan Cancer and Infectious Diseases Center Komagome Hospital, Tokyo, Japan; 13 Department of Orthopedic Surgery, Sagamihara National Hospital, Kanagawa, Japan; 14 Department of Orthopedic Surgery, Tokyo Metropolitan Tama Medical Center, Tokyo, Japan

**Keywords:** Outcomes research, Neurological disorders

## Abstract

The impact of body mass index (BMI) on outcomes after lumbar spine surgery is currently unknown. Previous studies have reported conflicting evidence for patients with high BMI, while little research has been conducted on outcomes for underweight patients. This study aims to examine the impact of BMI on outcomes after lumbar spine surgery. This prospective cohort study enrolled 5622 patients; of which, 194, 5027, and 401 were in the low (< 18.5 kg/m^2^), normal (18.5–30), and high (≥ 30) BMI groups, respectively. Pain was assessed via the numerical pain rating scale (NPRS) for the lower back, buttock, leg, and plantar area. Quality of life was assessed via the EuroQol 5 Dimension (EQ-5D) and Oswestry Disability Index (ODI). Inverse probability weighting with propensity scores was used to adjust patient demographics and clinical characteristics between the groups. After adjustment, the 1-year postoperative scores differed significantly between groups in terms of leg pain. The proportion of patients who achieved a 50% decrease in postoperative NPRS score for leg pain was also significantly different. Obese patients reported less improvement in leg pain after lumbar spine surgery. The outcomes of patients with low BMI were not inferior to those of patients with normal BMI.

## Introduction

Obesity, defined as a body mass index (BMI) ≥ 30 kg/m^2^^1^, is linked to spinal disorders (e.g., lower back pain^2^, disc degeneration^3,4^, vertebral fractures^5^, and complications of spine surgery)^6–9^. However, the impact of BMI on pain or other patient outcomes [e.g., physical function, quality of life (QoL)] after lumbar spine surgery remains unclear because previous studies have reported conflicting results^10–13^. One possible explanation for these conflicting results is that obese patients tend to have poorer preoperative functional scores compared to nonobese patients, which may provide them with greater opportunity for postoperative improvement^12^. Few published studies have analyzed outcomes after spinal surgery by statistically adjusting for the demographics and clinical characteristics of obese and nonobese patients.

While frailty or sarcopenia has been actively studied in recent years^14–18^, the impact of low BMI (< 18.5) on spinal surgery outcomes is even less well studied and understood than obesity. The lack of research involving underweight patients in spinal surgery may be related to the lesser prevalence of underweight people compared to obese people in the West. However, underweight individuals are relatively common in Japan. A 2019 survey conducted by the Japanese Ministry of Health, Labor and Welfare found that 8% and 4.6% of respondents were underweight and obese, respectively^19^. Therefore, the impact of low BMI on outcomes following spinal surgery may be particularly relevant in Japan and similar populations.

This study aims to investigate the association between BMI and patient-reported outcomes. Specifically, patient outcomes at 1 year following spinal surgery were compared in three distinct groups (low, normal, and high BMI) while statistically adjusting for patient demographics and characteristics.

## Materials and methods

### Patients

This study prospectively enrolled patients undergoing lumbar spine surgery in 13 hospitals in the Tokyo metropolitan area between April 1, 2017, and June 30, 2020. Patients were eligible for inclusion if they were underdoing a posterior spine surgery between L1 and S1 to treat a degenerative disease (e.g., lumbar spinal canal stenosis (LSS) or disc hernia). Patients were excluded if they were undergoing surgery with a planned dural incision; surgery for the treatment of a tumor, fracture, or infection; surgery using an anterior approach; if they did not agree to participate in this surveillance; or if the preoperative questionnaire was inadequately responded to (e.g., blank, illegible, or invalid answers). This study was approved by the Institutional Review Board of the Clinical Research Support Center of the University of Tokyo Hospital (approval no. 10335) and the Institutional Review Boards of all participating hospitals. All study procedures were conducted following the relevant guidelines and principles of the Declaration of Helsinki. A signed informed consent was obtained from all participants to participate in writing, including their consent to publish, and to withdraw from the study at any time.

### Background and surgical data

Clinical features, e.g., age, sex, BMI, diabetes mellitus (DM), hemodialysis (HD), rheumatoid arthritis (RA), smoking habits, usage of oral steroids, American Society of Anesthesiologists physical status classification (ASA-PS), and type of disease (e.g., LSS, disc hernia), were preoperatively collected for all patients. Operative factors, e.g., the type of surgical procedure (e.g., laminectomy/laminoplasty, herniotomy, and posterior fixation/fusion), operative time, estimated blood loss, primary, or revision surgery, microendoscopic surgery (yes/no), and occurrence of an unplanned dural tear during surgery (yes/no) were also noted.

### Clinical outcomes

Patient-reported outcomes were assessed before and 1 year after surgery. Pain was assessed using the Numeric Pain Rating Scale (NPRS) from 0 to 10 for the lower back, buttock, leg, and plantar area. QoL was assessed using the EuroQol 5 Dimension (EQ-5D) and the Oswestry Disability Index (ODI). Postoperative patient satisfaction was also evaluated at 1 year after surgery using a 7-point Likert scale (very satisfied, satisfied, slightly satisfied, neither satisfied nor dissatisfied, slightly dissatisfied, dissatisfied, and very dissatisfied)^20–22^. Patients were considered satisfied if they reported being very satisfied, satisfied, or slightly satisfied; patients reporting any other response were considered as dissatisfied.

### Statistical analysis

To investigate the association of BMI with patient-reported outcomes, all patients were classified into one of three groups: low (< 18.5), normal (18.5–30), or high (≥ 30) BMI groups. We used inverse probability weighting to adjust for differences in demographic and clinical characteristics between groups^23^. We chose this method over regression analysis because it allows for a more straightforward comparison between the three groups. First, a multinomial logistic model was used to calculate propensity scores (i.e., the probability that a patient belongs to a particular group)^24^. The model used age, sex, disease type, ASA-PS, DM, HD, RA, smoking habits, primary or revision surgery, and surgical procedure. Next, the weighted groups were created using the inverse probability weighting method with stabilized weights from propensity scores. Background data and clinical characteristics were compared using chi-square tests and one-way analysis of variance (ANOVA) for categorical and continuous variables, respectively. The differences concerning pre- and postoperative NPRS, ODI, and EQ-5D scores were examined by one-way ANOVA. Previous literature suggests that a 50% reduction in pain can be considered substantially important for patients with chronic pain^25^. Thus, a chi-square test was used to compare the rate of achieving this threshold between groups. Bonferroni corrections and Tukey’s multiple comparisons tests were used for all comparisons of categorical and continuous variables, respectively. In the Bonferroni corrections, each *p* value was tripled and the significance threshold was set to be the same as in other analyses.

To examine the nonlinear trend between BMI and NRS in more detail, restricted cubic spline logistic regression analysis was performed. Logistic regression analysis was performed for unweighted patients to calculate the OR of not achieving a 50% reduction in NRS using the same variables as inverse probability weighting with the addition of BMI. Patients with a BMI score of 25.0 were defined as the reference^26^.

All statistical analyses were performed using IBM SPSS Statistics 22.0 (IBM Corporation, Armonk, NY, USA). A restricted cubic spline was drawn using an open-source tool (https://mathworks.com/matlabcentral/fileexchange/41241-restricted-cubic-spline) using MATLAB R2020a (MathWorks, Inc., Natick, MA, USA). *P* values < 0.05 were considered statistically significant.

### Ethical approval

The manuscript submitted does not contain information about medical device(s)/drug(s).

## Results

### Patient background of unweighted groups

**Figure 1:**
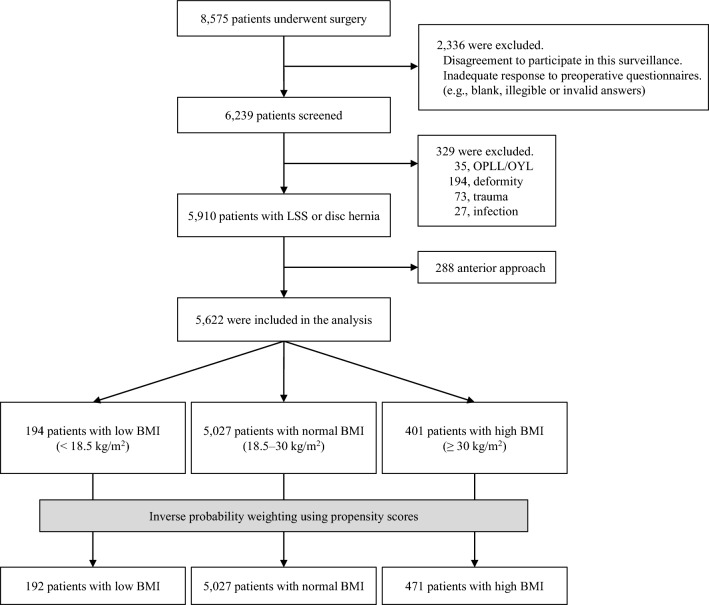
Flowchart of all the study population. *OPLL* Ossification of posterior longitudinal ligament, *OYL* ossification of yellow ligament, *LSS* lumbar spinal canal stenosis, and *BMI* body mass index.

Of all 8575 consecutive patients who underwent lumbar surgery, this study enrolled 5622 patients of whom 194 (3.5%), 5027 (89.4%), and 401 (7.1%) were in the low, normal, and high BMI groups, respectively (Fig. 1). Table 1 shows the patient background of unweighted groups. The proportion of males was lower in the group L (25.8%) than in the groups N (63.6%) and H (65.3%).

**Table 1:** Comparison of patient backgrounds and operative factors in the low, normal, and high BMI groups.

Variables	Low BMI (BMI < 18.5)		Normal BMI (18.5 ≤ BMI < 30)		High BMI (BMI ≥ 30)		P
No. of patients (%)	194	(3.5)	5027	(89.4)	401	(7.1)	
Mean age in years (SD)	62.7	(18.9)	64.6	(15.3)	57.3	(15.5)	< 0.001
Males, no. (%)	50	(25.8)	3198	(63.6)	262	(65.3)	< 0.001
Mean height in cm (SD)	158.6	(9.7)	162.5	(10.0)	163.8	(11.5)	< 0.001
Mean BMI in kg/m^2^ (SD)	17.5	(0.9)	24.0	(2.7)	33.2	(3.2)	< 0.001
Disease, no. (%)							
LSS	121	(62.4)	3532	(70.3)	272	(67.8)	0.042
Disc hernia	73	(37.6)	1495	(29.7)	129	(32.2)	
ASA-PS, no. (%)							
1	65	(33.5)	1196	(23.8)	29	(7.2)	< 0.001
2	107	(55.2)	3433	(68.3)	287	(71.6)	
3	22	(11.3)	396	(7.9)	85	(21.2)	
4	0	(0.0)	2	(0.0)	0	(0.0)	
Diabetes mellitus, no. (%)	17	(8.8)	734	(14.6)	114	(28.4)	< 0.001
Hemodialysis, no. (%)	9	(4.6)	96	(1.9)	9	(2.2)	0.029
Rheumatoid arthritis, no. (%)	5	(2.6)	74	(1.5)	5	(1.2)	0.421
Smoking, no. (%)	18	(9.3)	630	(12.5)	74	(18.5)	< 0.001
Steroid use, no. (%)	6	(3.1)	134	(2.7)	13	(3.2)	0.752
Surgery procedure, no. (%)							
Laminectomy	60	(30.9)	2171	(43.2)	180	(44.9)	0.006
Herniotomy	69	(35.6)	1414	(28.1)	121	(30.2)	
Post. fixation	65	(33.5)	1442	(28.7)	100	(24.9)	
Revision surgery, no. (%)	19	(9.8)	580	(11.5)	45	(11.2)	0.747
Microendoscopic, no. (%)	92	(47.4)	2325	(46.3)	190	(47.4)	0.869
Mean OT in minutes (SD)	124.8	(82.1)	130.5	(84.5)	149.3	(114.1)	< 0.001
Mean EBL in mL (SD)	141.4	(300.1)	140.9	(252.0)	188.2	(302.3)	0.002

BMI, Body mass index; SD, Standard deviation; LSS, Lumbar spinal canal stenosis; ASA-PS, American Society of Anesthesiologists physical status classification; OT, Operative time; EBL, Estimated blood loss.

### Inverse probability weighting and background of weighted groups

Following the inverse probability weighting using propensity scores, 192, 5027, and 471 patients were in the low, normal, and high BMI groups, respectively (Table 2). The mean and standard deviation (SD) of BMI was 17.4 (0.8), 24.0 (2.7), and 32.2 (2.6) in the low, normal, and high BMI groups, respectively. Significant differences were observed between groups in terms of mean age, mean height, ASA-PS, mean operative time, mean estimated blood loss, and the rate of unintended dural tear.

**Table 2:** Comparison of patient backgrounds and operative factors in the low, normal, and high BMI groups after adjustment by inverse probability weighting method.

Variables	Low BMI (BMI < 18.5)		Normal BMI (18.5 ≤ BMI < 30)		High BMI (BMI ≥ 30)		P
No. of patients (%)	192	(3.4)	5027	(88.3)	471	(8.3)	
Mean age in years (SD)	65.2	(18.5)	64.0	(15.5)	66.5	(14.1)	< 0.001
Males, no. (%)	118	(61.5)	3139	(62.4)	287	(60.9)	0.789
Mean height in cm (SD)	163.0	(9.8)	162.5	(10.0)	161.1	(11.2)	0.003
Mean BMI in kg/m^2^ (SD)	17.4	(0.8)	24.0	(2.7)	32.2	(2.6)	< 0.001
Disease, no. (%)							
LSS	135	(70.3)	3512	(69.9)	312	(66.2)	0.257
Disc hernia	57	(29.7)	1515	(30.1)	159	(33.8)	
ASA-PS, no. (%)							
1	44	(22.9)	1154	(23.0)	157	(33.3)	< 0.001
2	133	(69.3)	3422	(68.1)	283	(60.1)	
3	15	(7.8)	449	(8.9)	31	(6.6)	
4	0	(0.0)	2	(0.0)	0	(0.0)	
Diabetes mellitus, no. (%)	29	(15.1)	777	(15.5)	75	(15.9)	0.955
Hemodialysis, no. (%)	6	(3.1)	102	(2.0)	9	(1.9)	0.561
Rheumatoid arthritis, no. (%)	2	(1.0)	75	(1.5)	4	(0.8)	0.479
Smoking, no. (%)	29	(15.1)	648	(12.9)	51	(10.8)	0.274
Steroid use, no. (%)	3	(1.6)	136	(2.7)	20	(4.2)	0.088
Surgery procedure, no. (%)							
Laminectomy	85	(44.3)	2158	(42.9)	192	(40.8)	0.264
Herniotomy	53	(27.6)	1433	(28.5)	157	(33.3)	
Post. fixation	55	(28.6)	1436	(28.6)	122	(25.9)	
Revision surgery, no. (%)	23	(12.0)	574	(11.4)	46	(9.8)	0.527
Microendoscopic, no. (%)	85	(44.3)	2338	(46.5)	230	(48.8)	0.502
Mean OT in minutes (SD)	124.7	(78.1)	129.9	(83.8)	144.5	(89.5)	0.002
Mean EBL in mL (SD)	133.7	(260.1)	139.8	(250.4)	173.8	(282.0)	0.020

BMI, Body mass index; SD, Standard deviation; LSS, Lumbar spinal canal stenosis; ASA-PS, American Society of Anesthesiologists physical status classification; OT, Operative time; EBL, Estimated blood loss.

### Clinical outcomes in the weighted groups

Table 3 shows the comparison of each clinical outcome between the three weighted groups. The NPRS was completed by 69.4% of patients in the study (Table 3). The groups differed significantly in terms of preoperative lower back pain (low BMI group, 4.7 [SD 3.1]; normal BMI group, 5.5 [3.0]; and high BMI group, 6.4 [3.0]; *p* < 0.001) and buttock pain (low BMI group, 4.0 [3.4]; normal BMI group, 5.0 [3.4]; and high BMI group, 4.9 [3.3]; *p* = 0.002). Pairwise comparisons between groups revealed that preoperative lower back pain in the high BMI group was worse than in the low and normal BMI groups (both *p* < 0.001). Preoperative buttock pain was worse in the low BMI group compared to the normal (*p* < 0.001) and high BMI groups (*p* = 0.024). Postoperative NRPS scores differed significantly in terms of leg pain (low BMI group, 2.0 [2.6]; normal BMI group, 2.4 [2.8]; high BMI group, 3.0 [3.2]; *p* < 0.001). Pairwise comparisons between groups demonstrated that postoperative leg pain in the high BMI group was worse than in the low and normal BMI groups (*p* = 0.001 and *p* < 0.001, respectively).

**Table 3:** Comparison of NRS, EQ-5D, ODI, and postoperative satisfaction in the low, normal, and high BMI groups after adjustment by inverse probability weighting method.

Variables		Low BMI (BMI < 18.5)		Normal BMI (18.5 ≤ BMI < 30)		High BMI (BMI ≥ 30)		p	p		
									L vs. N	L vs. H	N vs. H
NPRS, no. of patients (%)		121	(3.1)	3460	(87.7)	366	(9.3)				
Lower back, mean (SD)	Pre	4.7	(3.1)	5.5	(3.0)	6.4	(3.0)	< 0.001	0.088	< 0.001	< 0.001
	Post	2.6	(2.6)	2.9	(2.6)	2.9	(2.9)	0.524			
Buttock, mean (SD)	Pre	4.0	(3.4)	5.0	(3.4)	4.9	(3.3)	0.002	0.001	0.024	0.712
	Post	1.3	(2.3)	1.5	(2.4)	1.5	(2.6)	0.551			
Leg, mean (SD)	Pre	5.8	(3.4)	6.1	(3.1)	5.8	(3.5)	0.138			
	Post	2.0	(2.6)	2.4	(2.8)	3.0	(3.2)	< 0.001	0.153	0.001	< 0.001
Plantar, mean (SD)	Pre	2.2	(2.9)	2.3	(3.0)	1.9	(3.0)	0.076			
	Post	1.4	(2.3)	1.3	(2.3)	1.2	(2.2)	0.934			
Achievement of 50% relief, no. (%)											
Lower back		72	(59.5)	1955	(56.5)	229	(62.6)	0.072			
Buttock		94	(77.7)	2653	(76.7)	283	(77.5)	0.912			
Leg		93	(76.9)	2324	(67.2)	218	(59.6)	< 0.001	0.077	0.002	0.010
Plantar		84	(69.4)	2504	(72.4)	275	(75.1)	0.391			
EQ-5D, no. (%)		128	(3.2)	3564	(88.1)	354	(8.7)				
	Pre	0.54	(0.20)	0.54	(0.17)	0.55	(0.14)	0.599			
	Post	0.73	(0.18)	0.75	(0.19)	0.75	(0.20)	0.096			
	Post–Pre	0.19	(0.27)	0.21	(0.23)	0.20	(0.22)	0.136			
ODI, no. (%)		124	(3.2)	3434	(87.9)	349	(8.9)				
	Pre	44.0	(19.4)	44.0	(18.9)	46.9	(16.6)	0.047	0.989	0.304	0.037
	Post	21.4	(16.7)	19.5	(18.0)	21.2	(19.1)	0.176			
	Post–Pre	− 22.6	(20.1)	− 24.4	(21.8)	− 25.7	(22.2)	0.358			
Satisfaction, no. (%)											
Satisfied		114	(89.1)	2836	(82.1)	296	(82.7)	0.126			
Not satisfied		14	(10.9)	618	(17.9)	62	(17.3)				

BMI, Body mass index; SD, Standard deviation; NPRS, Numerical pain rating scale; EQ-5D, EuroQol 5 Dimension; ODI, Oswestry disability index.

The proportion of patients who achieved a 50% decrease in postoperative NPRS score differed significantly in terms of leg pain (low BMI, 76.9%; normal BMI, 67.2%; and high BMI, 59.6%; *p* = 0.001). Pairwise comparison between groups showed that the proportion was lower in the high BMI group than in the normal (*p* = 0.010) and low BMI group (*p* = 0.002). The proportion appeared better, but was not significant (*p* = 0.077), in the low BMI group than in the normal BMI group.

Of the 5690 patients enrolled in the study, 4046(completion rate: 71.1%; 128, 3,564, and 354 in the low, normal, and high BMI groups) and 3,907 (completion rate: 68.7%; 124, 3,434, and 349 in the low, normal, and high BMI groups) completed the EQ-5D and ODI, respectively (Table 3). No significant differences were noted between groups in either pre- or postoperative EQ-5D scores. On the contrary, a significant difference was observed between groups in preoperative ODI (low BMI group, 44.0 [19.4]; normal BMI group, 44.0 [18.9]; and high BMI group, 46.9 [16.6]; *p* = 0.047). The high BMI group demonstrated a worse ODI score than the normal BMI group in the pairwise comparisons (*p* = 0.037). However, the change in preoperative to 1-year postoperative score in terms of EQ-5D and ODI were not significantly different between groups (EQ-5D, *p* = 0.136; ODI, *p* = 0.358).

Patient satisfaction data were collected from 3,940 (69.2%) patients (Table 3). No significant difference in postoperative satisfaction was observed among the three weighted groups (satisfaction rates: low BMI group, 89.1%; normal BMI group, 82.1%; and high BMI group, 82.7%; *p* = 0.126).

### Restricted cubic spline logistic regression analysis

**Figure 2:**
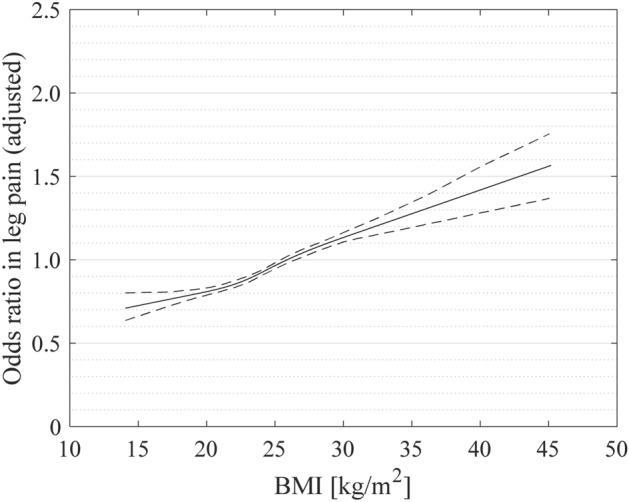
A restricted cubic spline (solid line) shows adjusted odds ratio for non-achieving a 50% reduction in leg pain after lumbar spine surgery, drawn with four knots of body mass index (BMI) percentiles 5 (19.0 kg/m^2^), 35 (22.8 kg/m^2^), 65 (25.4 kg/m^2^), and 95 (31.0 kg/m^2^), with 25.0 kg/m^2^ as the reference. Smoothed dotted lines indicate 95% confidence intervals.

Figure 2 shows the results of restricted cubic spline logistic regression analysis for leg pain, wherein the proportion of patients achieving a 50% reduction in postoperative NRS score was found to be significant. This shows that the OR of not achieving a 50% reduction in leg pain increased as BMI increased (Fig. 2).

## Discussion

### Leg pain in obese patients

In this study, the percentage of patients achieving a 50% reduction in leg pain worsened as BMI increased. Thus, patients with lower BMI had greater odds of achieving a 50% reduction in leg pain than patients with higher BMI. The relationship between BMI and leg pain remains controversial. Djurasovic et al. retrospectively reviewed patients undergoing lumbar fusion (*N* = 270) and reported that obese patients had slightly higher leg pain scores two years after surgeries than nonobese patients (mean NPRS 5.10 vs. 4.29; *p* = 0.043)^27^. De la Garza-Ramos et al. also investigated the impact of obesity on patients who underwent one- to three-level posterolateral fusion for degenerative spine disease^28^. They found that a higher proportion of obese patients had radiculopathy after surgery than nonobese patients (44.3% [31/70] vs. 30.4% [201/662]; *p* = 0.018). However, Brennan et al. reported no significant difference in pre and postoperative (3 or 12 months) leg pain scores between obese and nonobese patients who underwent lumbar discectomy (*N* = 107)^29^. These discrepancies may be partially explained by differences in patient demographics and baseline characteristics or by small sample sizes. The results of the current study may be more reliable due to the large sample size and statistical analysis, which adjusted for the potentially confounding differences in demographics and preoperative characteristics between groups.

Since many studies define a BMI score ≥ 30 as obesity, the cutoff value was set to 30^30^. What outcomes are exhibited by those with a BMI score of 25–30 (overweight) and ≥ 40 (super-obese) would be interesting to find out, but the stratification used in this study does not allow us to see this. Therefore, we drew a restricted cubic spline and showed that as BMI increases, the OR of not achieving a 50% reduction in leg pain also increases. Hence, we conclude that at least for leg pain post lumbar spine surgery, the lower the BMI, the better the postoperative outcome.

One possible reason that obese patients are less likely to experience a significant reduction in leg pain after spinal surgery could be related to cytokines secreted from excess adipose tissue (i.e., adipokines)^31–35^. In obese patients, increased secretion of proinflammatory cytokines and decreased secretion of anti-inflammatory cytokines from adipose tissues have been observed, which can lead to increased levels of proinflammatory cytokines (tumor necrosis factor-α, interleukin 6, and so on) and systemic inflammation^35^. Inflammation can cause peripheral and central sensitization of the pain-transmitting system, resulting in hyperalgesia and allodynia^35^. Visceral and subcutaneous truncal white adipose tissue, often found in obese patients, is an active endocrine organ that secretes cytokines^33^, which may affect decompressed nerve roots and prolong pain in obese patients.

### Oswestry disability index scores in obese patients

Patients in the high BMI group were observed to have worse preoperative ODI scores than patients with normal BMI and higher levels of back pain than patients in the low and normal BMI groups. Although obese people tend to have worse back pain and ODI scores than nonobese people^26,27,36^, a controversy exists regarding the impact of obesity on ODI score improvement after lumbar spine surgery^11–13,26,27,37^. Djurasovic et al. reported no significant difference in improvement between obese and nonobese patients in ODI scores two years after lumbar fusion surgery (mean change, 14.03 vs. 15.35; *p* = 0.602)^27^. Additionally, Rihn investigated the Spine Patient Outcomes Research Trial dataset and found that obese patients with LSS showed equivalent outcomes to nonobese patients after posterior decompressive laminectomy with or without fusion in terms of their postoperative ODI improvement [mean change after 1 (21.7 vs. 20.8; *p* = 0.63) and 4 (17.6 vs. 20.1; *p* = 0.22) years]^37^. Conversely, Knutsson et al. reported from the Swedish Spine Register that obese patients had inferior ODI scores compared to nonobese patients after any surgery for LSS^26^. However, postoperative improvement in ODI, EQ-5D, and patient satisfaction was found in this study to not significantly differ between obese and nonobese patients. These results suggest that obese patients should expect similar levels of improvement in QoL as nonobese patients.

### Patient-reported outcomes in underweight patients

Patients in the low BMI group were similar to patients in the normal BMI group in terms of postoperative satisfaction and postoperative improvement of NPRS, ODI, and EQ-5D. Although several studies related to complications for underweight patients seeking lumbar spine surgery were noted, only a few reports are available on their postoperative outcomes relative to patients with normal or high BMI. Knutsson et al. stratified 2633 patients who underwent lumbar surgery for LSS in the Swedish Spine Register into three groups: underweight/normal patients (BMI < 25), overweight patients (BMI of 25–30), and obese patients (BMI ≥ 30). The investigators examined differences between these groups 2 years after surgery using the visual analog scale (VAS) for back pain and leg pain and the EQ-5D and ODI for QoL^26^. They found no significant differences between underweight/normal patients and overweight patients in terms of back pain (mean VAS, 31 vs. 33; *p* = 0.20), leg pain (mean VAS, 31 vs. 32; *p* = 0.54), or EQ-5D (mean, 0.64 vs. 0.63; *p* = 0.63). The underweight/normal group had better ODI scores compared to overweight patients (mean, 25 vs. 27; *p* = 0.01). The investigators also performed a restricted cubic spline logistic regression analysis to calculate an OR for dissatisfaction. They found that patients with a BMI of 15–25 had an OR of almost 1.0 (i.e., as likely to be satisfied as those with BMI = 25). The results of the current study are consistent with the results of Knutsson et al.^26^.

Based on previous literature and this current study, it appears that lumbar spine surgery outcomes for patients with low BMI may not be inferior to patients with normal BMI.

### Limitations

This study has several limitations. First, the sample size of the low and high BMI groups was smaller than the normal BMI group. Second, the completion rate for each clinical outcome was not high, which could have biased the results. Third, detailed surgical variables (e.g., the number of operated disc levels, the type of implant used, or years of surgeon experience) were not adjusted. These variables may differ between groups and could have unknowingly impacted the results of the current study. Fourth, knowing why a given patient has a high or low BMI is difficult. BMI can be affected by genetics, lifestyle, exercise habits, or various diseases (diabetes, heart failure, chronic kidney disease, liver dysfunction, lung disease, cancer, and so on). Patients who are underweight due to their lifestyle are likely to differ in important ways from patients who are underweight secondary to cancer. Furthermore, data related to frailty or sarcopenia were not collected. Fifth, although related to the third limitation, some selection bias may exist regarding which patients undergo surgery. Some obese and underweight patients may be unable to undergo surgery due to their underlying diseases that impact their BMI. Unfortunately, this potential selection bias was not tracked or accounted for before the decision to undergo surgery. Finally, a detailed image evaluation, which could have been useful to further adjust for baseline differences between groups, was not conducted.

## Conclusion

In this study, patients in the high BMI group had worse postoperative leg pain and were less likely to experience significant leg pain improvement than patients in the low and normal BMI groups. Patients in the high BMI group also had worse preoperative ODI scores, but demonstrated similar postoperative ODI improvement compared to patients in the low and normal BMI groups. Patients in the low BMI group demonstrated similar postoperative satisfaction and improvements in NPRS, ODI, and EQ-5D compared to patients in the normal BMI group.

## Data Availability

The datasets generated and/or analyzed during the current study are available from the corresponding author on reasonable request.
